# Genome editing in large animals: current status and future prospects

**DOI:** 10.1093/nsr/nwz013

**Published:** 2019-01-31

**Authors:** Jianguo Zhao, Liangxue Lai, Weizhi Ji, Qi Zhou

**Affiliations:** 1 State Key Laboratory of Stem Cell and Reproductive Biology, Institute of Zoology, Chinese Academy of Sciences, Beijing 100101, China; 2 Savaid Medical School, University of Chinese Academy of Sciences, Beijing 100049, China; 3 Institute of Stem Cell and Regeneration, Chinese Academy of Sciences, Beijing 100101, China; 4 South China Institute for Stem Cell Biology and Regenerative Medicine, Guangzhou Institutes of Biomedicine and Health, Chinese Academy of Sciences, Guangzhou 510530, China; 5 Yunnan Key Laboratory of Primate Biomedicine Research, Institute of Primate Translational Medicine, Kunming University of Science and Technology, Kunming 650500, China; 6 CAS Center for Excellence in Brain Science and Intelligence Technology (CEBSIT), Shanghai 200031, China

**Keywords:** genome editing, large animal, disease model, agriculture, xenotransplantation

## Abstract

Large animals (non-human primates, livestock and dogs) are playing important roles in biomedical research, and large livestock animals serve as important sources of meat and milk. The recently developed programmable DNA nucleases have revolutionized the generation of gene-modified large animals that are used for biological and biomedical research. In this review, we briefly introduce the recent advances in nuclease-meditated gene editing tools, and we outline these editing tools’ applications in human disease modeling, regenerative medicine and agriculture. Additionally, we provide perspectives regarding the challenges and prospects of the new genome editing technology.

## INTRODUCTION

Animal models are indispensable for understanding disease pathogenesis, and developing novel therapeutic agents and treatments [1]. Genetically modified classical model organisms (e.g. nematodes, fruit flies, zebrafish and rodents) have provided a vast array of experimental data, resulting in new-found knowledge and insights to advance our understanding of human biology and disease. However, researchers still have concerns about the ability of model organisms to represent the complex spectrum of human biology and the relevance of the findings for translation to humans [2,3]. Thus, there is a demand for alternative and optimized models that are more evolutionarily similar to humans, or that more adequately recapitulate human physiological characteristics, both in health and disease. By improving the availability and quality of optimized animal models, the translation of preclinical studies in model organisms to clinical trials in humans might become more of a reality.

Large animals, especially non-human primates (NHPs), pigs and dogs, share many similarities with humans: physiology, organ size, anatomy and metabolism [2,4–6]. These similarities make them ideal organ donors for xenotransplantation and excellent models for human diseases, such as neurodegenerative disease (ND) and cardiovascular disease. With the identification of mutations responsible for human diseases, targeted modification of these genes in large animals provides useful disease models for pathology studies, drug discovery, and development and regenerative medicine research. In pigs and dogs, genetic modification has been very slow and tedious because of the lack of characterized embryonic stem cells (ESCs), the extremely low efficiency of homologous recombination (HR) and the time consuming breeding programs required to obtain biallelic genetically modified animals. The progress of genetic modification in NHPs is also slow going since zygote injection, combined with inefficient HR, limits the possibility to achieve precise and tailored editing. A recent NHP cloning platform was successfully established in February 2018 and no genetically modified animals have been reported by this strategy so far [7]. Fortunately, we have witnessed the rapid development of nuclease-mediated genome editing technology, which has revolutionized the production of genetically modified large animals, resulting in dramatically increased numbers of genetically modified animals.

During early domestication, large livestock animals were first genetically modified using conventional breeding and selection to produce improved livestock. Although modern transgenic processes have also been used to produce livestock, these high-profile studies are typically proof-of-principle studies, and commercial application of genetically modified animals is still limited and under development. However, the recently developed nuclease-mediated genome editing technology, which dramatically improves the spectrum for making genetic modifications in livestock, has stimulated interest in the generation and use of genome-edited livestock. With the new livestock technologies, beneficial alleles that would otherwise be lost in conventional breeding could be conserved using the novel genome editing tools, and also result in reduced cost and a shortened timeframe for generating the desired mutant animals. Furthermore, precision editing in the endogenous genome, without introducing foreign DNA, could become a new breeding technology to produce genetically modified organisms for human consumption.

In this review, we briefly provide an overview of the development of nuclease-mediated gene editing tools, and discuss the representative studies that have been achieved using gene editing tools for modeling human diseases, regenerative medicine and agricultural applications.

## NUCLEASE-MEDIATED GENOME EDITING

Several nucleases have been successfully used for gene editing, including zinc finger nuclease (ZFN), transcription activator-like effector nuclease (TALEN), and the clustered regularly interspaced short palindromic repeat (CRISPR) and CRISPR-associated (Cas) protein 9 system. Over HR-based conventional gene targeting, all these nuclease-based gene editing tools enable site-directed genome engineering with tremendous advantages, including efficiency, low cost and simplicity, etc. A new era has arrived for genetic modifications, especially in large animals, for biological and biomedical investigation.

The first synthetically engineered, genome editing agents were ZFNs, which combine the binding module zinc finger protein (ZFP) with the restriction enzyme domain FokI (an endogenous restrictive endonuclease from *Flavobacterium okeanokoites*). For genome editing, a pair of ZFPs need to bind regions flanking the target locus to form a FokI dimer, which is necessary to induce double-strand breaks (DSBs) [8]. Similarly, TALENs are also modular proteins that contain two domains: a customizable DNA-binding domain (TALE) and a FokI nuclease domain. Dimerized FokI cuts TALE-binding DNA sequences, thereby producing DSBs in a similar way to ZFNs [9].

TALEN-mediated gene editing was selected by the scientific society as one of the top 10 scientific breakthroughs in 2012, and both ZFNs and TALENs have been successfully used to generate genetically modified large animals [10–12]. However, due to the extensive protein–DNA contacts of ZFNs and the highly repetitive nature of TALENs, targeting of different sites in the genome by ZFNs and TALENs required the re-design or re-engineering of a new set of proteins. The difficulty in cloning and protein engineering ZFNs and TALENs partially prevented these tools from being broadly adopted by the scientific community [13–15]. In this respect, CRISPR has revolutionized the field because it is as robust as, if not more so than, the existing tools in terms of editing efficiency. More importantly, it is much simpler and more flexible to use. With significant technical barriers for ZFNs and TALENs, the CRISPR system has dominated the genome editing field since 2013.

### CRISPR-based genome editing

**Figure 1.**
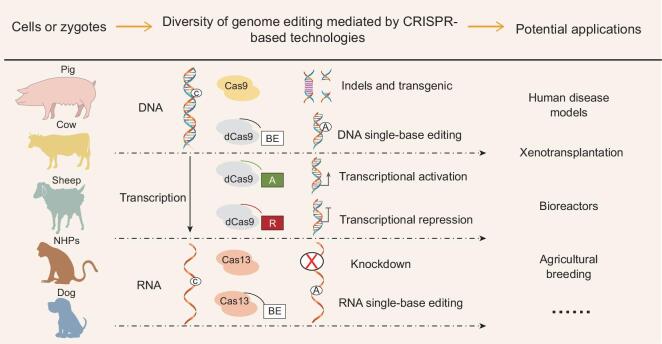
Major strategies to recruit DNA- and RNA-targeting and modifying enzymes via the CRISPR/Cas systems, and their potential applications in large animals to life science fields. Left panel: large animals including pig, cow, sheep, monkey and dog are discussed in this review. Middle panel: CRISPR-based technologies have been developed to edit DNA and RNA, and regulate transcription. Right panel: potential applications of genome-edited large animals in modeling human diseases, offering xenotransplant organs, livestock breeding and more.

CRISPR was first identified and characterized as an unexpected defense mechanism used by bacteria to fight off viruses in 2007 [16]. In 2012, the function of Cas protein was first demonstrated to cleave specific DNA sequences, guided by short synthetic pieces of RNA *in vitro* [17]. *In vivo* demonstrations followed in 2013, with the laboratories of Church, Doudna and Zhang quickly exhibiting the power of CRISPR-mediated genome modification in mammalian cells [18–20]. Due to their scalability, affordability, and engineering flexibility, CRISPR/Cas technologies fueled biological and biomedical investigations in multiple cell types and living organisms, through a variety of efficient and versatile genetic modifications, such as deletions, knockins, RNA regulation and chromatin modification of targeted gene loci (Fig. 1) [18]. This innovative genome editing tool has created a paradigm shift in the life sciences, and was selected as ‘Science's 2015 Breakthrough of the Year’ [16].

The CRISPR/Cas system functions in three phases successively: adaptation, expression and interference. During adaptation, short pieces of foreign DNA are captured and integrated as ‘spacer’ elements into the CRISPR loci. Then, the assembled CRISPR locus (spacers separated by repeat regions) is transcribed to yield a pre-CRISPR RNA (crRNA), which is processed to generate crRNA. The crRNA identifies and guides Cas effector proteins to disrupt the target sequences [21–23]. The CRISPR/Cas9 system has only one effector protein, Cas9, and harbors two functional units, HNH and the RuvC-like domain. The HNH nuclease domain cleaves the complementary strand to the crRNA-guide sequence, whereas the Cas9 RuvC-like domain cleaves the non-complementary strand. The dual trans-activating crRNA:crRNA binds to the target genomic locus adjacent to a protospacer motif (PAM)-NGG, and the nuclease generates site-specific DSBs, highlighting the potential to exploit the system for RNA-programmable genome editing [17,18,24]. The simple and effective introduction of DSBs makes CRISPR/Cas9 a very powerful tool compared with ZFNs and TALENs, offering an unprecedented range of targets in a large variety of functional domains within various genomic sites. With the goal of versatile genome editing and fewer off-target effects, several natural and engineered CRISPR nucleases have been developed, including SaCas9, Cpf1 and XCas9, which have been fully reviewed [13,25].

Of note, the binding capacity of the CRISPR/Cas9 system inspired geneticists to develop transcriptionally regulated systems based on dCas9, a Cas9 whose endonuclease activity has been removed. As reviewed by the Adli group, dCas9-based tools have been well developed and employed in gene regulation, epigenetic regulation, chromatin imaging, chromatin topology and base editing [26]. Additionally, the newly developed RNA-targeting CRISPR-based tools, such as Cas13a, Cas13b, Cas13c and Cas13d, provide a platform for mRNA regulation that substantially extends the spectrum of CRISPR-mediated genome editing [27].

### Base editor systems

Most genetic diseases arise from point mutations; however, gene corrections achieved by conventional approaches (including ZFNs, TALENs and CRISPR/Cas) often induce an abundance of random insertions and deletions at the target locus because of the presence of DSBs. To circumvent this hurdle, a new approach to genome editing that enables the direct, irreversible conversion of one target DNA base into another in a programmable manner, without requiring dsDNA backbone cleavage, excess stochastic insertions and deletions, or dependence on homology-directed repair, was first reported by David Liu’s group in 2016. The base editor system directly deaminates a cytidine or adenine base [28].

The first-generation base editor system (BE1) was composed of dCas9 and a cytidine deaminase enzyme, which successfully converted cytidine into thymidine with a catalytic window of activity of −16 to −12 bp from the PAM sequence [28]. Subsequently, BE2 was developed by the addition of the uracil glycosylase inhibitor (UGI) to the C-terminus of BE1. Cytidine deaminase converts cytosine into uracil, and subsequently, uracil DNA glycosylase can perform error-free repair, converting the uracil into the wild-type sequence. The addition of the UGI inhibits the base-excision repair pathway, resulting in a 3-fold increase in efficiency [29]. Another major improvement of the system was achieved by the development of BE3, which uses the Cas9 D10A nickase and resulted in a 6-fold increase in base editing [30]. Then, three improvements were made for the generation of the BE4 system: extending the cytidine deaminase-Cas9n linker to 32 amino acids, extending the Cas9n-UGI linker to nine amino acids and appending a second copy of UGI construct BE4, which increased efficiency by approximately 50%, while halving the frequency of undesired byproducts compared to BE3 [31]. By modification of nuclear localization signals and codon usage, and ancestral reconstruction of the deaminase component, base editors BE4max and AncBE4max were optimized. The resulting BE4max editors correct pathogenic single-nucleotide polymorphisms (SNPs) with substantially increased efficiency in a variety of mammalian cell types [32]. Fusion with a rationally engineered SpCas9 variant (SpCas9-NG) that can recognize relaxed NG PAMs, and an activation-induced cytidine deaminase, mediates the conversion of cytidine into thymidine at target sites with NG PAMs in human cells [33].

Adenine base editors (ABEs) have also been generated to modify adenine bases [34,35]. The most active ABEs generated include ABE5.3 with an activity window of −3 to −6 bp from the protospacer, and ABE7.8, ABE7.9 and ABE7.10 with an activity window of −4 to −9 bp from the protospacer [35]. Additionally, modifications were made in ABEs to produce an ABE4max system for increased editing efficiency. The resulting ABE4max editors could correct pathogenic SNPs (convert A:T to G:C), which account for 61% of human pathogenic SNPs in the ClinVar database, with substantially increased efficiency in a variety of mammalian cell types compared with ABE7.10 [32].

Such base editor tools, which modify genes at the single-base level without causing DSBs, hold great promise for applications in basic biology, trait development in livestock and gene therapy in genetic diseases. However, some technical limitations, such as editing efficiency, accuracy, sequence spectrum and the window of activity of base editors, still need to be worked out and fine-tuned.

## GENETIC ENGINEERING IN LARGE ANIMALS FOR DISEASE MODELS

Most biological and biomedical experiments are performed on rodents, but complementary and validating preclinical evaluations are often performed on large animals to evaluate the relevance of findings for translation to humans. NHPs, pigs and dogs are generally accepted by researchers to be useful animals for human disease modeling, and have been widely used in biomedical research. The technological development of precision gene editing tools avoids some of the difficulties encountered with genome modification in large animals and facilitates the generation of genetically modified large animals that could be used for biomedical research.

### NHPs

NHPs, such as rhesus and cynomolgus monkeys, resemble humans in evolution, anatomy, physiology and pathology more than any other animal, which makes them the most attractive species for human disease models. A recent *SIRT6* deletion study in monkeys substantiates this concept. In mice and other lower organisms, *SIRT6* is identified to be a longevity protein that regulates many factors, such as genome stability, inflammation and metabolism, that are associated with ageing [36]. However, the NHP study challenged the mouse-based orthodoxy by demonstrating that CRISPR/Cas9-meditated SIRT6-deficient cynomolgus monkeys died shortly after birth and displayed severe global prenatal developmental retardation [37,38]. This study shows the huge differences of aging regulation between NHPs and rodents, and questions whether *SIRT6* is involved in longevity in humans.

The first NHP disease model was created in 2008 using lentivirus-mediated expression of the polyglutamine-expanded huntingtin gene (HTT) in rhesus macaques. In the transgenic monkeys, hallmark features in brain and behavioral defects, similar to those found in individuals with Huntington disease (HD), were observed, providing a valuable model for behavioral and cognitive assessment, disease progression and therapeutic development [39]. However, precise genetic modification was inconceivable before the emergence of nuclease-mediated genome editing. With the most abundant NHP resources in the world, Chinese scientists are leading the field of genome editing in NHP models. In 2014, Liu *et al.* first used TALENs to mutate the X-linked *methyl-CpG binding protein 2 (MECP2)* gene in rhesus and cynomolgus monkeys to model Rett syndrome (RTT). The male *MECP2* mutant fetuses in the cynomolgus monkeys exhibited mid-gestation lethality, which is consistent with RTT-linked male embryonic lethality in humans [40]. The female *MECP2* mutant monkeys exhibited physiological, behavioral and structural abnormalities, as well as immune gene dysregulation, which resembled the clinical manifestations of individuals with RTT [41]. Two years later using TALENs, Ke *et al.* successfully generated a biallelic *microcephalin 1* (*MCPH1*) mutant cynomolgus monkey that recapitulated most of the clinical characteristics observed in individuals with microcephaly: smaller head circumference, hypoplastic corpus callosum, premature chromosome condensation and behavioral abnormalities [42]. While TALENs initiated the movement to create mutant NHPs for modeling human diseases, CRISPR/Cas9 has exponentially sped up the innovation of new nuclease-meditated gene editing tools and subsequent NHP models.

Multiple monkey models were developed by the CRISPR/Cas9 system. *PPARγ* (*peroxisome proliferator- activated receptor gamma*) and *RAG1* (*recombination activating gene 1*) double-mutant cynomolgus monkeys were first created without detecting off-targeting mutagenesis [43,44]. Next, Wan *et al.* injected an optimized CRISPR/Cas9 system into monkey zygotes, ultimately producing a p53 biallelic (homozygous) mutant monkey that offered a model to study tumorigenesis. This study also demonstrated the feasibility of generating homology-directed repair (HDR)-mediated precise nucleotide-substitution mutations in monkey embryos for the first time, suggesting the potential to create models that faithfully mimic human genetic mutations [45]. Another example is the Duchenne muscular dystrophy (DMD) monkey model, successful created by CRISPR/Cas9-mediated deletion of the *dystrophin* gene. The resulting monkeys exhibited early muscle degeneration, which could be used to develop therapeutic interventions at an early stage for this disease [46]. Another successful gene deletion was shown by Kang *et al*. They successfully knocked out *DAX1 (dosage-sensitive sex reversal, adrenal hypoplasia critical region, on chromosome X, gene 1)* in cynomolgus monkeys, and the *DAX1*-deficient monkey displayed an adrenal gland development defect, abnormal testis architecture and unaffected Sertoli cell formation. These *DAX1*-deficient monkey characteristics more faithfully resembled manifestations found in individuals with AHC-HH (adrenal hypoplasia congenital and hypogonadotropic hypogonadism) than *DAX1*-deficient mouse characteristics [47].

All of the described NHP models were created via microinjection, which often causes a high degree of mosaicism and limits the ability to obtain genetically uniform animals. In 2018, Liu *et al.* first successfully made two cynomolgus monkeys by somatic cell nuclear transfer (SCNT) [7]. By incorporating this delicate yet difficult procedure, researchers could circumvent some of the variability problems and accelerate the production of genetically uniform monkey models for human diseases. Together, the successful somatic cloning procedure and the rapidly developing genome editing system could be used to promote the development of tailored NHP models, and improve the feasibility of using NHP models for biomedical research in the future.

### Pigs

Pigs have been widely used in biomedical research over recent decades because of breeding and handling advantages, and fewer ethical concerns when compared with NHPs. Pigs have an early sexual maturity (5–8 months), a short gestation period (about 114 d), and delivery of multiple offspring (about 10–12 piglets per litter), making them a suitable species for preclinical experimentation. In addition, gene editing tools and pig SCNT are fully developed. Pigs with gene knockout have been consistently generated since 2002 [48]; however, progress has been very slow and a limited number of porcine models were created before the emergence of nuclease-mediated gene editing tools.

ZFNs were first applied to pigs with the knockout of the transgenic *enhanced green fluorescent protein* (*eGFP*) and *PPARγ* genes, proving the feasibility of nuclease-mediated gene editing in pigs [11,49]. With the development of TALENs and CRISPR/Cas9, the efficiency, throughput and precision in pig genetic modification was further accelerated, and more tailored models were generated. TALENs were used to generate *low density lipoprotein receptor (LDLR)* monoallelic and biallelic mutant Ossabaw pigs as models of familial hypercholesterolemia [50]. In Chinese Bama miniature pigs, zygote co-injection of Cas9 mRNA and sgRNA (single guide RNA) was used to delete *Npc1l1 (Niemann-Pick C1-Like 1)*, efficiently producing biallelic mutant pigs to study how Npc1l1 influences cardiovascular and metabolic diseases [51]. For a model to study the molecular mechanism of human atherosclerosis, *apolipoprotein E (ApoE)* and *LDLR* double-knockout pigs were created using CRISPR/Cas9. The *ApoE/LDLR* biallelic knockout pigs exhibited elevated levels of low density lipoprotein cholesterol (LDL-C), total cholesterol (TC) and apolipoprotein B in serum [52]. To study hypertrophic cardiomyopathy (HCM), Montag *et al.* successfully introduced the HCM point mutation (R723G) into the porcine *MYH7* gene using TALENs. The resultant heterozygous pigs displayed HCM phenotypes, including mild myocyte disarray, malformed nuclei and MYH7 overexpression. The early onset of HCM disease in these animals highlights the importance of using pigs to study the mechanisms and progression of human cardiac disease [53]. Hai *et al.* first applied the CRISPR/Cas9 system to pigs and generated vWF (von Willebrand Factor) biallelic mutants, which exhibited significantly reduced coagulation factor FVIII activity and a severe bleeding phenotype, consistent with von Willebrand disease (vWD) [54].

Animal models are important for screening drugs but the right animal model(s) need(s) to be selected. For example, only 5% of anti-cancer agents in preclinical development showed sufficient efficacy in phase III testing, although they showed efficacy in mouse models of cancer [55]. Cancers in mice are biologically different from humans and findings based on murine models often do not translate into the clinic. Thus, animal models that are more representative of the human cancer spectrum are in great demand. The immune system in pigs shares similarities with humans for more than 80% of the analyzed parameters, whereas mice are similar to humans in less than 10% [56,57]. This might make pigs a more suitable animal model for human cancers. The first nuclease-mediated porcine cancer model was created using TALENs to introduce the *adenomatous polyposis coli (APC)* heterozygous mutation, providing a colon cancer model [58]. Then, He *et al.* created *PKD1* (polycystic kidney disease 1) monoallelic knockout pigs using ZFNs; the resultant pigs exhibited renal cysts at 6 months that progressively grew, providing a good model for studying renal cystogenesis [59]. Wang *et al.* established a pROSA26-iCas9 pig line with Cre-inducible Cas9 expression using TALENs, which allowed *ex vivo* and *in vivo* genome modifications. By delivering Cre recombinase and sgRNAs targeting multiple cancer gene loci to the lungs, F1 pRosa26-iCas9 pigs grew large primary lung tumors and showed lung cancer symptoms. These pigs with primary tumors will provide a new platform to develop models of human cancer, possibly facilitating new diagnostic and therapeutic technologies [60].

NDs are another disease that would benefit from new diagnostic and therapeutic technologies. Although NDs affect a large number of people of all ages, no effective therapies have been developed for the vast majority of NDs. Because the brain structure of a pig is more similar to that of a human than a rodent, a number of pig models have been generated to study NDs. Three pig lines that model Parkinson’s disease (PD) were created using TALENs and CRISPR/Cas9:*DJ1* knockout, *PARK2/PINK1* double knockout or *Parkin/DJ-1/PINK1* triple knockout. These pigs served as models for PD pathology studies and therapeutic intervention development [61–63]. Mutant pigs have also been used to try to create behavioral and neuropsychiatric disorder models. By deleting tryptophan hydroxylase-2 (*TPH2*) with CRISPR/Cas9, pigs displayed dramatically reduced levels of serotonin (5-HT), and impaired survival and growth rates before weaning [64]. Recently, a Huntingtin (HTT) knockin pig model of HD was created using CRISPR/Cas9, and the resultant pigs exhibited movement and behavioral abnormalities, and selective degeneration of striatal medium spiny neurons at early stages, which recapitulated the selective neurodegeneration of individuals with HD perfectly [65]. Together, these studies provide strong evidence that pigs can be used to model NDs.

Pigs are also good animals to model skin diseases. The structure of pig skin, including thickness, the dermal–epidermal interface, hair follicle content, pigmentation, collagen and lipid composition, and dermal blood, is very similar to the structure of human skin [66,67]. Because of these similarities, several genes associated with pigmentation or skin disease were modified using nuclease-mediated genome editing to create disease models. When tyrosinase (*TYR*) was biallelically mutated with CRISPR/Cas9, typical albinism was observed in the mutant pigs, including pigment loss in the skin, hair and eyes [62]. Another pig line that shows albinism was developed by Wang *et al.* These *MITF* biallelic mutant pigs have a white coat color phenotype, clinical manifestations and underlying causal genetics of human Waardenburg syndrome [68]. Pigs have also been used to model ectodermal dysplasia-9 (ED-9) by deleting *Hoxc13* with CRISPR/Cas9. The resulting *Hoxc13*-knockout pigs exhibited external hair loss, reduced hair follicles and abnormal hair follicle structure, but normal skin structure, skeleton phenotype and growth, which is consistent with the phenotypes of individuals with ED-9 [69].

In summary, with the development of nuclease-mediated genome editing technology, the generation of porcine mutants to model human diseases has greatly expanded, and will help the understanding of pathogenesis progression and therapeutic development for human diseases.

### Dogs

Dogs, like monkeys and pigs, share many metabolic, physiological and anatomical characteristics with humans. Interestingly, more than 450 canine hereditary diseases provide naturally occurring disease models, and about half of these afflicted dogs exhibit striking clinical similarities that correspond to human disease, which can be investigated to study disease pathogenesis and preclinical treatment [6]. Naturally occurring hereditary disease phenotypes in dogs have also contributed to the discovery of genes and genetic pathways associated with disease, such as DMD [70,71]. However, few genetically modified dog models have been created because of unique species-specific reproductive characteristics. Zou *et al.* first produced *MSTN* (myostatin) biallelic knockout dogs via zygote injection of Cas9 mRNA and sgRNA combined with autologous embryo transfer. One of the resulting dogs exhibited a double-muscle phenotype of the thighs at 4 months, which demonstrated the feasibility of generating dog models for biomedical research [72]. Another dog model created with genome editing technology was an atherosclerotic cardiovascular disease model incorporating an ApoE biallelic mutation [73]. In 1-month-old dogs, plasma LDL, TC and triglyceride (TG) levels were all elevated, suggesting that dogs could be used to study disease progression and screen new drugs to treat human atherosclerotic disease. Contrarily, dog models have also been used to suggest that gene editing approaches could be clinically useful. In a DMD dog model, the expression of the dystrophin gene was successfully restored by systemic delivery of CRISPR gene editing components. The dogs showed improved muscle histology, demonstrating the potential application of gene editing approaches in the treatment of individuals with DMD [74].

**Figure 2.**
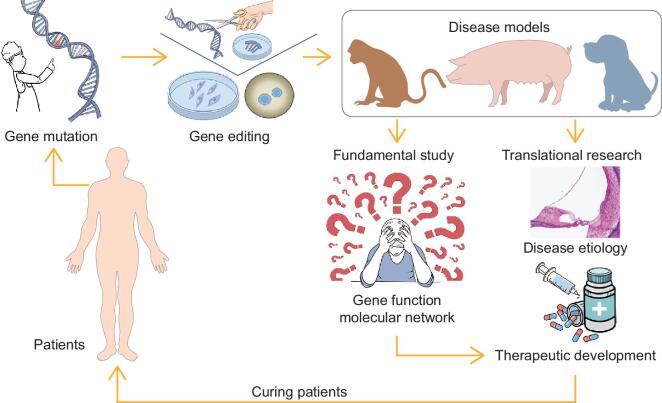
Disease models using large animals contribute to basic science as well as translational science research. Nuclease-mediated genome editing can introduce specific mutations into orthologous genes in large animals, including NHPs, pigs and dogs. Large animal disease models contribute to our fundamental understanding of the underlying mechanisms of disease and, therefore, therapeutic development to treat or cure human disease.

With the technical evolution of genome editing tools, more and more disease models in large animals have been created, providing valuable opportunities to study the mechanisms of human diseases and to further develop therapeutic interventions (Fig. 2). An ideal animal model for human diseases should replicate the complex phenotypes of human diseases to the largest extent and also the underlying causality. Considering this, choosing a large animal species in place of a rodent model for disease modeling is scientifically justifiable. Comparative pathology and feasibility will be central to choosing the appropriate animal models for a specific application. However, there is growing social aversion to the use of dogs and NHPs as laboratory animals, and the experimental use of NHPs is tightly regulated in Europe and the USA. Even with these hurdles, NHPs are indispensable for certain biomedical research, such as ND and vaccine development. For other studies, the features of the pig, mentioned above, combined with an increasing availability of biological tools and reagents for studying porcine tissue, make the pig arguably the best and most feasible model available for translational biomedical research.

## XENOTRANSPLANTATION

Organ transplantation provides a very promising solution for patients suffering from end-stage organ failure; however, the severe shortage of human organs has led to a major transplantation crisis. According to the latest statistics, 300 000 patients in China are estimated to need a transplant, but only about 16 000 operations are likely to be performed each year [75]. Xenotransplantation could provide a solution where the transplantation of animal cells, tissues or organs could replace an injured tissue or whole organ in humans. In fact, the first blood transfusions were xenotransfusions, carried out in the 17th century [76]. In the 20th century, a number of NHP organ xenotransplantations were attempted [77]. In 1963–1964, 13 recipients were transplanted with chimpanzee kidneys, and one of them returned to work for almost 9 months before suddenly dying [77,78]. In 1964, the first experimental clinical trial of chimpanzee-to-human heart transplantation was carried out, but the patient died within 2 h [79]. Two years later, the first chimpanzee-to-human liver transplantation was carried out by Starzl, but the patient never recovered [75]. In 1992, a patient survived for 70 d after transplantation with a baboon liver and immunosuppression [80,81]. While these studies raise feasibility concerns, other hurdles for NHP xenotransplantation are risk of the organ infection, time, the expense of NHP breeding and the limited availability of NHP organs [82].

Pigs, as organ donors for humans, have a number of advantages. They share a number of anatomical and physiological similarities, have organs of comparable size and are cost-effective in breeding [83]. Despite these advantages, pig-to-human xenotransplantation has two major hurdles: immune rejection and potential cross-species infection, such as via Porcine Endogenous Retrovirus (PERV). In recent decades, these two hurdles have been partially overcome by incorporating various genetic modifications in pigs and by using recent sophisticated genome editing tools. These strategies further promote the possibility of bringing pig-to-human xenotransplantation to the hospital.

### Pigs genetically engineered for compatibility with the human immune system

Despite pig organs serving as major candidates for human transplantation, pig organs generally, and unsurprisingly, trigger significant immune rejection responses in humans, resulting in complete failure of the transplanted organs. Hyperacute rejection (HAR) develops immediately after transplantation, mainly caused by binding of preexisting antibodies in human plasma to the galactosyl-α(1,3) galactose (Gal) epitope on the surface of swine endothelial cells. Because the Galα(1,3)Gal epitope is biosynthetically generated by the enzyme α1,3-Galactosyltransferase (GGTA1), removing GGTA1 from pigs could be the first step toward overcoming HAR. In 2002, pigs were produced with heterozygously and homozygously inactivated GGTA1 [84,85]. Using hearts from GGTA1-knockout (GTKO) pigs, xenotransplantation resulted in one graft surviving 6 months [86]. To further decrease the xenoreactive process in HAR, Lutz *et al.* created pigs lacking CMAH (encoding cytidine monophosphate-N-acetylneuraminic acid hydroxylase) and β4GalNT2 (encoding β1,4-N-acetylgalactosaminyltransferase), and these double knockout pigs showed a decreased humoral barrier to xenotransplantation compared with pigs lacking only GGTA1 [87]. Moreover, CRISPR/Cas9-edited pigs that lacked GGTA1, CMAH and β4GalNT2 genes had less human immunoglobulin (Ig)M and IgG binding *in vitro* to their PBMCs than pigs that lacked only GGTA1 and CMAH [88].

Another vital factor leading to hyperacute rejection is complement activation, and several human complement regulatory proteins (e.g. CD46, CD55 and CD59) have been identified as promising targets to reduce complement activity in xenotransplantation. In fact, organs from transgenic pigs with human hCD59 have been shown to be protected from complement attack [89]. Moreover, kidneys from human CD55 transgenic pigs combined with an immunosuppressive strategy have also been shown to result in the longest survival time (∼78 d) when transplanted into bilaterally nephrectomized cynomolgus monkeys (*Macaca fascicularis*) [90]. Finally, heart transplants from hCD46 transgenic pigs to baboons combined with anti-pig antibody inhibitors led to a median graft survival of up to 96 d [91]. In 2005, transgenic pigs were produced that expressed all three human complement factors: hCD46, hCD55 and hCD59. Cytotoxicity assays indicated that they would provide greater protection against complement activity than either hCD59 or hCD55 alone [92]. Remarkably, the longest surviving xenograft (> 900 d) was seen with a pig-to-baboon heart transplant, using GTKO pigs that expressed hCD46 and human thrombomodulin (GTKO/hCD46/hTBM) [93].

Additionally, physiological incompatibilities during xenotransplantation can activate a blood-mediated inflammatory reaction (IBMIR), giving rise to coagulative disorders [94]. In an attempt to avoid IBMIR, many genetically modified pigs were generated that incorporated genes related to the human coagulation system. For example, pig models expressing human CD39 (platelet aggregation genes), tissue factor pathway inhibitor and thrombomodulin (an inhibitor of factors Va and VIIIa) have demonstrated coagulation inhibition [95–97].

Another major hurdle to successful xenograft survival is the xenogenic cellular response, such as that resulting from differences between porcine (swine leukocyte antigens) and human (human leukocyte antigens, HLA) major histocompatibility complexes [98]. Cytotoxicity against porcine tissues is mainly mediated by natural killer cells and T lymphocytes, and can be alleviated by transgenic expression of HLA-E in porcine endothelial cells [99,100]. Significantly, additional expression of HLA-E in GTKO/hCD46 pigs enhanced median lung survival (> 4 h), attenuated the rise in pulmonary vascular resistance, and reduced platelet activation and histamine release [101]. In addition, Ide *et al.* demonstrated that transgenic expression of hCD47 in porcine cells decreased their susceptibility to macrophage phagocytosis *in vitro* [102]. A final strategy used to inhibit xenograft rejection includes introducing anti-inflammatory and anti-apoptotic genes, such as the human heme oxygenase 1 (HO-1) gene, which protects cells against apoptosis and inflammation, and the human zinc finger protein A20 gene, which inhibits the activity of NF-κB and TNF-mediated programmed cell death. Transgenic expression of these two genes conferred potential protection against xenograft rejection [103,104].

**Figure 3.**
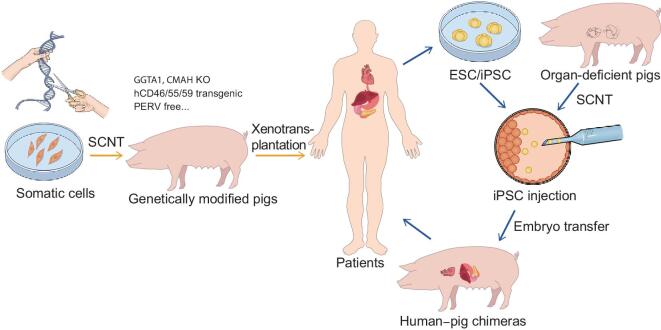
Strategies to manipulating pig organs to make them compatible to humans. Left panel: genetically modified pigs could provide organs compatible with the human immune system (e.g. *GGTA1*, *CMAH* knockout, hCD46, hCD55 and hCD59 transgenic) and free of PERV. Right panel: hESCs or iPSCs were injected into genetically modified pig embryos that lacked specific tissues or organs, generating human organs in the human–pig chimeras.

Collectively, extensive progress has been made with generating genetically modified pigs that are compatible with the human immune system, offering hope for cross-species transplantation (Fig. 3).

### Interspecies chimera generated by blastocyst complementation

An alternative strategy to produce functional and transplantable tissues or organs is to build an interspecies chimera at the embryonic level. During the early stage in embryonic development, a lack of commitment from progenitor cells can lead to an ‘empty’ developmental niche in the animal embryo. These empty niches can then be ‘filled’ with human ESCs (hESCs) or human induced pluripotent stem cells (hiPSCs) with chimeric capability, resulting in the generation of organs originating from human donor cells. In this regard, several organ-specific developmental genes that govern tissue and organ formation were disrupted, and ultimately led to the generation of embryos, fetuses or neonates that lacked entire tissues or organs [105]. Next, hESCs or hiPSCs were injected into these host blastocysts, leading to the production of human organs and a chimeric host [106]. The original proof-of-concept of chimeric hosts was shown with rodents. Wild-type mouse ESCs were injected into RAG-2 (recombination-activating gene 2) knockout recipient mouse blastocytes to rescue T- and B-cell development [107]. Mouse models lacking specific organs have been developed, including *Pdx1-*knockout mice (lacking pancreases) [108], *Sal1-*knockout mice (kidney agenesis) [109], *Runx1*-knockout mice (disrupted hematopoiesis) [110] and *Nkx2.5*-knockout mice (retarded cardiac development) [111]. The mice provide ‘empty’ developmental niches for performing blastocyst complementation. In 2007, wild-type mESCs were injected into *Pdx1-*knockout mouse embryos, rescuing the development of the defective pancreas [112]. A few years later, the first interspecies blastocyst complementation was performed, with rat pluripotent stem cells (PSCs) being injected into *pdx1*-deficient mouse blastocysts to successfully generate a rat pancreas in mice [113]. Vice versa, injected mouse PSCs in a *Pdx1-*deficient rat model generated a mouse pancreas in rats, which kept host blood glucose levels at a normal range for > 370 d [114]. Using a similar strategy, Isotani *et al.* successfully generated a functional rat thymus using rat ESCs in nude mice [115]. In spite of the remarkable low degree of chimerism, human–mouse interspecies chimeras could also be created by injecting human naive PSCs into mouse preimplantation embryos [116,117]. Those human–mouse studies inspired many scientists to explore the possibility of generating allogeneic or xenogeneic organs in large animals, which could be used for transplants. In 2013, Hiromitsu Nakauchi's group demonstrated that the blastocyst complementation system could generate functional pancreata using wild-type blastocystmeres with pancreatogenesis-disabled Pdx1-Hes1 host embryos [118]. Moreover, Wu *et al.* demonstrated the feasibility of creating chimeric embryos between humans and large domestic animals, including pigs and cattle. The group also found an intermediate hPSC type, which exhibits a higher degree of chimerism in postimplantation pig embryos [119]. All these studies suggest the dawning of a new era for transplantation, providing a future possibility of generating human organs in pigs.

### Pigs genetically engineered for removing threatening viruses

Another major obstacle to implanting pig organs into humans is the widespread presence of PERV. These viruses are remnants of ancient viral infections and are harmless in pigs, but they could potentially become activated and affect a human recipient of a porcine allograft. Many strategies to prevent PERV transmission have been developed, including vaccination [120,121] and RNA interference [122–125], but ultimately they only reduced the expression of PERV. ZFN-mediated gene editing also failed when being used to eliminate PERVs from the pig genome [126]. Exciting results were provided by Yang *et al.*, who succeeded in inactivating 62 copies of proviruses in the pig genome. The group used CRISPR/Cas9 in a pig cell line and finally used SCNT to produce PERV-inactivated pigs, offering a glimmer of hope for clinical application of PERV-free pig organs in xenotransplantation [127,128].

## GENOME EDITING IN LIVESTOCK FOR AGRICULTURAL BREEDING

Darwin clearly pointed out that both nature and artificial selection have shaped animal and plant breeds: ‘The key is man's power of cumulative selection: nature gives successive variations; man adds them up in certain directions useful to himself’ [129]. In comparison, natural selection tends to be driven by many genes (i.e. polygenic adaptation), while artificial selection is often based on a handful of genes with a large effect size.

Over the last 50 years, many sophisticated breeding procedures have been developed in quantitative genetics to select animals with outstanding breeding values [130]. However, artificial selection has to rely on natural variation, and selection of a favorable genotype is often associated with loss of genetic variability, inbreeding depression and sometimes deleterious alleles. In addition, breeding experiments with livestock are often painstakingly slow and costly. Thus, innovations in breeding strategies are expected to significantly improve livestock production.

In the past few years, the development of nuclease-mediated gene editing technologies has revolutionized the field of livestock breeding. Since large animal genomes can be modified efficiently, it is not surprising that many more animals with elite phenotypes have been produced in the last few years, using genome editing, compared with the previous three decades. Potential agricultural benefits of these livestock include lactation performance, meat production, disease resistance and bioreactors, which cannot be easily achieved with conventional breeding procedures.

### Milk modification

β-Lactoglobulin (BLG) is a major whey protein that is the dominant allergen in milk from goats, cows and other ruminants (normally absent in human milk), and can cause allergy symptoms ranging from mild to life-threatening. Heat processing and enzymatic hydrolysis are two commonly used methods to reduce the allergenic potential of BLG, but these biochemical approaches are costly and may affect milk nutritive value by producing unexpected by-products. Genetic modification could be a more direct approach to reduce BLG levels in ruminants’ milk, and both ZFNs and TALENs have been used to mutate BLG in cattle [131,132]. In the TALEN system, cattle with the BLG mutation were free of any mature BLG [132]. In addition, BLG is also an ideal locus in mammary gland bioreactors, and the human lactoferrin (hLF) gene was knocked in using TALENs in goats. Phenotyping in the goats revealed large-scale hLF expression and the absence of BLG in milk [133]. Using the same strategy, Luo *et al.* obtained high expression levels of human serum albumin in the milk of cows [134]. Using these accurate genome editing strategies, more pharmaceutical proteins are expected to be produced in livestock milk in the future.

### Meat production, composition and quality

Myostatin (MSTN) is a protein secreted in muscle tissues and its primary function is to negatively regulate muscle growth. The natural mutation of MSTN leads to a double muscle trait, first reported in cattle and then in sheep, dogs and humans [135], making *MSTN* an attractive target for genome editing to increase lean meat in livestock. In 2015, a ZFN-mediated *MSTN*-mutation in Chinese Meishan pigs led to developmentally normal animals that exhibited an increase in muscle mass by 100% and a decrease in fat accumulation compared with wild-type animals [136]. Enhanced body weight and larger muscle fiber size were also observed in goats with disrupted MSTN [137]. In goats, the MSTN locus was also used to insert the *fat-1* gene, which converts n-6 polyunsaturated fatty acid (PUFA) to n-3 PUFA. The genetically modified goats had improved muscle growth performance and also produced healthier meat by decreasing the ratio of n-6 PUFA to n-3 PUFA, which has been reported to be a risk factor for many life-threatening diseases [138].

Genome editing technology has also been used to enhance livestock thermoregulation. Pigs lack functional uncoupling protein (UCP1), which makes them cold intolerant and prone to fat deposition, causing neonatal death and lower production efficiency. *UCP1* localizes on the inner mitochondrial membrane, can generate heat by uncoupling ATP synthesis from proton transit across the inner membrane, and is likely the most important regulator in body thermoregulation and adiposity. In pigs, CRISPR/Cas9 was used to insert mouse adiponectin-UCP1 into the endogenous *UCP1* locus, and the UCP1-knockin pigs showed an improved ability to maintain body temperature during acute cold exposure with normal physical activity. The UCP1-knockin pigs showed increased lean meat and decreased fat deposition compared with control pigs, making them a valuable resource for the pig industry [139]. This study highlights the potential of employing biotechnology in pig breeding to improve quantitative traits.

Another pig trait that could be improved is that pigs cannot efficiently digest their food, leaving excessive nutrients such as phosphorus and nitrogen to be released into the environment. To address the issue of environmental emissions in the pig industry, transgenic pigs harboring a single-copy quad-cistronic transgene were created that expressed three microbial enzymes—beta-glucanase, xylanase and phytase—in their salivary glands. In pigs expressing the three enzymes, digestion of non-starch polysaccharides and phytate in the feed was enhanced. The transgenic pigs also had decreased fecal nitrogen and phosphorus outputs, and increased growth rates and feed conversion rates (by 11.5–14.5%) compared with that of age-matched wild-type littermates given the same feed. These findings suggest that transgenic pigs could be promising resources for improving feed efficiency and reducing the environmental impact of pigs [140].

### Disease resistance

Porcine reproductive and respiratory syndrome (PRRS) is the most economically devastating disease affecting industrial swine worldwide. Vaccines have been developed against the PRRS virus (PRRSV), but they provide poor swine protection due to the genetic diversity of the virus. The cellular receptor for the PRRSV has been identified as CD163, a cellular protein in the scavenger receptor cysteine-rich (SRCR) superfamily, making the receptor a potential target to block PRRSV infection. With the aid of the CRISPR-Cas9 system, *CD163*-null pigs were quickly generated. In the *CD163*-null pigs that were exposed to PRRSV or infected penmates, no viremia or clinical signs were observed [141], providing proof-of-concept that a single-gene deletion establishes PRRSV-resistant pig breeds. Inspired by this, further CRISPR/Cas9 precision editing was performed by either deleting SRCR domain 5 or by replacing the domain with the human orthologous CD163 domain (domain swap) [142,143]. These studies demonstrated that the SRCR 5 domain was the interaction site for the virus.

Foot-and-mouth disease virus (FMDV) is also another economically devastating viral disease facing the swine industry worldwide. Transgenic pigs were generated that constitutively expressed FMDV-specific short interfering RNAs derived from small hairpin RNAs (shRNAs) and transgenic pigs exposed to the virus displayed no clinical signs of viral infection when compared with wild-type pigs, offering another example of genetic engineering for disease resistance [144].

Bovine tuberculosis, which is caused by *Mycobacterium bovis*, is becoming a serious threat to the agricultural economy and global public health (transmission from cattle to humans) [145]. Currently, no effective programs exist to eliminate or control bovine tuberculosis. One gene of interest is the natural resistance-associated macrophage protein-1 gene (*NRAMP1*), which is also known as the solute carrier family 11A member 1 gene (*SLC11A1*). The gene has been found to be associated with innate resistance to intracellular pathogens such as *Mycobacterium*, *Leishmania*, *Salmonella* and *Brucella*, and the resistance is suspected to be induced by multiple proinflammatory responses. Indeed, transgenic cows with a site-specific NRAMP1 insertion confirmed the function of NRAMP1 in providing resistance to tuberculosis [146]. In addition, the mouse SP110 gene emerged as a promising candidate to control infection by *M*. *bovis* by limiting its growth in macrophages and inducing apoptosis in infected cells. With TALEN-mediated insertion of the mouse SP110 gene into the cattle genome, transgenic cattle were capable of controlling the growth of *Mycobacterium* and limiting the transmission of tuberculosis in penmates [147].

### Animal welfare

Physical dehorning of cattle is used to protect animals and producers from accidental injury, but is costly and painful for the animals. Genetic analyses have identified variants that are associated with cattle hornlessness (i.e. ‘polled’), a trait that is common in beef but rare in dairy breeds. Fewer beef cattle than dairy cattle need to be dehorned because the dominant POLLED locus is nearly fixed in beef cattle, such as Angus. Dairy breeds, such as Holstein, have a much lower frequency of POLLED with only a small number of sires (6%) producing commercially available POLLED semen. Thus, a candidate ‘polled’ allele was introgressed into dairy cattle using TALEN-mediated genome modification and reproductive cloning. Hornless dairy cattle were obtained, providing evidence for genetic causation and a means to introduce polled’ into livestock with the potential to improve the welfare of millions of cattle without crossing [148].

### Bioreactors

Livestock have also been used as bioreactors to produce human biological products. Blood-derived human serum albumin (HSA) is prescribed for a number of severe diseases, such as liver failure and traumatic shock, and is in high demand. Due to the shortage of human blood supplies and the infection risks associated with human blood, alternative ways to produce HSA have long been sought. Success was found when CRISPR/Cas9 was used to knock in human albumin cDNA to the pig endogenous albumin locus, leading to transgenic piglets with human albumin in their blood [149].

**Figure 4.**
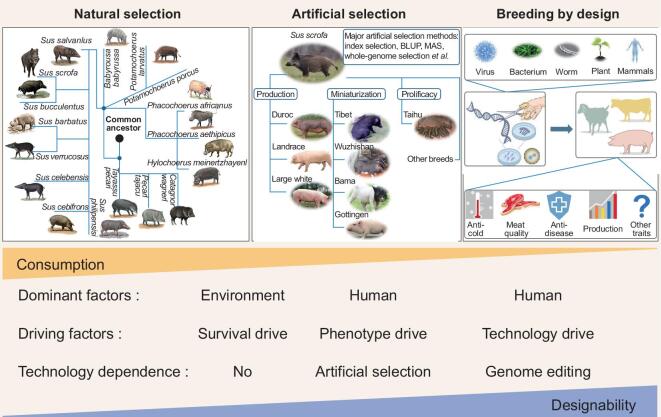
CRISPR/Cas system-mediated genome editing in farm animals has created a new era of breeding by design. Left panel: suiforme diversity and phylogenetic relationship of *Sus scrofa*. With the power of natural selection, *S*. *scrofa*, *S*. *salvanlus et al*. evolved from the same ancestor [156]. Pig pictures are adapted from the animal diversity website at the University of Michigan Museum of Zoology. Middle panel: driven by artificial selection, including index selection, best linear unbiased prediction (BLUP), marker-assisted selection (MAS) and whole-genome selection, pig breeds with advanced production ability (Doruc, Landrace and Large white), miniaturized body (Tibet, Wuzhishan and Bama) and high prolificacy (Taihu) were created. Right panel: with the aid of CRISPR/Cas-mediated genome editing, functional genes or loci from viruses, bacteria, worms, plants and other elite animals are able to be introduced into livestock for designed traits. Evidence is presented for the feasibility of breeding by design, such as thermoregulation, meat quality, disease resistance and livestock production. Breeding mediated by genome editing dramatically improves the spectrum for making genetic modifications in livestock, and reduces the costs and timeframes for generating desired mutant animals.

In summary, genome-edited livestock can be produced more efficiently and precisely, and advanced editing tools offer much promise in accelerating genetic improvements in farm animals. To meet these expectations, more genomic markers controlling genetic variation or causative genes in economically important livestock phenotypes will need to be revealed, facilitated by robust genome editing technology. That is to say, nuclease-mediated genome editing technologies will arm researchers with powerful new tools to improve farm animal breeding, leading them to a new area of ‘*in vitro* breeding’ or ‘breeding by design (Fig. 4).

## PROSPECTS AND CHALLENGES

Genome editing technology provides revolutionary ways to change, regulate, determine and imagine genomes in large animals, potentially offering novel applications in biomedicine and agriculture. We anticipate greater numbers of applications materializing in the near future, such as genome-edited NHPs combined with SCNT, pig organ xenotransplantation used in clinical trials, and genome-edited livestock-derived meat making its way to the food table. However, challenges still remain with integrating genome editing into biomedicine and agriculture. It is obvious that the effectiveness and specificity of genome editing with currently available tools still needs improvement, and that the safety and ethical concerns of using genetically modified tissues, organs and animals remain a focus of considerable debate. Specifically, a strategy to generate large founder animals with a desired allele in one step, without a prolonged period of breeding, is in high demand. However, mosaic mutations, which are commonly observed in zygote injection-based genome editing, are another potential challenge in the editing of large animals. These issues could potentially be solved by tagging Cas9 with ubiquitin-proteasomal degradation signals [150] and introducing editing components in an appropriate format (i.e. a Cas9 protein/sgRNA complex) into very early-stage zygotes [151,152]. Other possible strategies to reduce mosaicism have been discussed in a recent review [153].

Gene therapy, using genetic modification with exogenous DNA to treat inherited human diseases, offers new treatment modalities in multiple medical fields. Currently, three gene therapy products have been approved by the US Food and Drug Administration (FDA): LUXTURNA™ (manufactured by Spark Therapeutics, Inc.) for the treatment of patients with confirmed biallelic RPE65 mutation-associated retinal dystrophy, KYMRIAH™ (manufactured by Novartis Pharmaceuticals Corporation) for the treatment of patients up to 25 years of age with B-cell precursor acute lymphoblastic leukemia (ALL) that is refractory or in second or later relapse, and YESCARTA™ (manufactured by Kite Pharma, Inc.) for the treatment of adult patients with relapsed or refractory large B-cell lymphoma after two or more lines of systemic therapy. In addition, the European Medicines Agency has approved Glybera for lipoprotein lipase deficiency and the FDA has assigned LentiGlobin BB305 as a breakthrough therapy designation request for treatment of transfusion-dependent patients with β-thalassemia major. However, a broader spectrum of somatic cell editing needs to be developed, both *ex vivo* and in humans. Animal models that match and recapitulate the characteristics of human disease are a top priority for evaluating the efficacy and safety of gene therapy or cell-based therapy to treat human disease.

In spite of the substantial potential of genome editing for clinical and agricultural applications, safety and ethical issues cannot be ignored. Xenotransplantation provides hope to patients living with organ failure and waiting for a donor, yet the use of animal organs and tissues in humans is still not fully accepted due to safety and ethical concerns. Further confirmation of the efficacy and safety of xenotransplantation is urgently needed for the procedure to gain acceptance. On the other hand, with the advent of interspecies chimeras using blastocyst complementation, human organs may one day be produced in large animals. However, researchers and the public still have concerns about the risk of human cells integrating into the host animal's brain or germline, and these concerns need to be taken into account. Unlike transgene technology, which introduces an exogenous gene into the host genome randomly, genome editing only changes the endogenous gene in an efficient and accurate way. With these new technologies, the FDA is maintaining a product-focused, science-based regulatory policy with specific legal standards applied to different types of products. The FDA has determined that animals with intentionally altered genomes should be subjected to regulations under the provisions of new animal drugs [154]. Unlike the FDA, the US Department of Agriculture (USDA) has stated that the USDA will not regulate genetically modified plants produced by the new genome editing techniques [155], which will definitely accelerate the commercialization of genome-edited organisms. With further studies to solve the ‘off-target’ effects and potential risks to the host genome, genome editing of animals may become more accepted by the public.

In summary, the rapid progress of genome editing in large animals has resulted in the production of many valuable animals for human disease models, xenotransplantation and the agricultural economy (Table 1). Further optimization of the existing genome editing system and the generation of new tools for precise gene modification will additionally accelerate the development of genetically modified animals, organs and tissues for agriculture, regenerative medicine and therapeutic applications.

**Table 1.** List of the genetically modified animals mentioned above.

Species	Gene	Modifications	Approach	Applications	References
Rhesus	HTT	Transgenic	Random integration	Disease model for HD	[39]
Rhesus/ cynomolgus	MECP2	KO	TALENs	Disease model for RTT	[40]
Cynomolgus	MECP2	KO	TALENs	Disease model for RTT	[41]
Cynomolgus	MCPH1	KO	TALENs	Disease model for microcephaly	[42]
Cynomolgus	PPARγ/RAG1	KO	CRISPR/Cas9	Disease model for metabolic diseases and immunodeficiency	[43,44]
Cynomolgus	p53	KO	CRISPR/Cas9	Disease model for studying tumorigenesis	[45]
Rhesus	Dystrophin	KO	CRISPR/Cas9	Disease model for DMD	[46]
Cynomolgus	DAX1	KO	CRISPR/Cas9	Disease model for AHC-HH	[47]
Pig	PPARγ	KO	ZFNs	Disease model for metabolic diseases	[11]
Pig	LDLR	KO	TALENs	Disease model for familial hypercholesterolemia	[50]
Pig	Npc1l1	KO	CRISPR/Cas9	Disease model for cardiovascular and metabolic diseases	[51]
Pig	ApoE/LDLR	KO	CRISPR/Cas9	Disease model for cardiovascular diseases	[52]
Pig	MYH7	Point mutation KI	TALENs	Disease model for HCM	[53]
Pig	vWF	KO	CRISPR/Cas9	Disease model for vWD	[54]
Pig	APC	KO	TALENs	Disease model for colon cancer	[58]
Pig	PKD1	KO	ZFNs	Disease model for renal cystogenesis	[59]
Pig	TP53/PTEN/APC/ BRCA1/BRCA2/KRAS	KO/point mutation	CRISPR/Cas9	Disease model for lung cancer	[60]
Pig	DJ-1	KO	TALENs	Disease model for PD	[61]
Pig	PARK2/PINK1	KO	CRISPR/Cas9	Disease model for PD	[62]
Pig	Parkin/DJ-1/PINK1	KO	CRISPR/Cas9	Disease model for PD	[63]
Pig	TPH2	KO	CRISPR/Cas9	Disease model for 5-HT deficiency induced behavior abnormality	[64]
Pig	Huntingtin	KI	CRISPR/Cas9	Disease model for HD	[65]
Pig	TYR	KO	CRISPR/Cas9	Disease model for albinism	[62]
Pig	MITF	KO	CRISPR/Cas9	Disease model for Waardenburg syndrome	[68]
Pig	Hoxc13	KO	CRISPR/Cas9	Disease model for ED-9	[69]
Dog	MSTN	KO	CRISPR/Cas9	Improve muscle growth, new strains	[72]
Dog	ApoE	KO	CRISPR/Cas9	Disease model for cardiovascular disease	[73]
Dog	Dystrophin	KO	CRISPR/Cas9	DMD gene therapy	[74]
Pig	GGTA1	KO	HR	Xenotransplantation	[84,85]
Pig	CMAH/ β4GalNT2	KO	ZFNs	Xenotransplantation	[87]
Pig	GGTA1/CMAH/ β4GalNT2	KO	CRISPR/Cas9	Xenotransplantation	[88]
Pig	hCD46/hCD55/ hCD59	Transgenic	Random integration	Xenotransplantation	[92]
Pig	GGTA1/hCD46/ hTBM	KO and transgenic	HR/random integration	Xenotransplantation	[85,93]
Pig	hCD39	Transgenic	Random integration	Xenotransplantation	[95]
Pig	Thrombomodulin	Transgenic	Random integration	Xenotransplantation	[97]
Pig	GGTA1/hCD46/ HLA-E	KO and transgenic	HR/random integration	Xenotransplantation	[85,101]
Pig	hCD47	Transgenic	Random integration	Xenotransplantation	[102]
Pig	hHO-1	Transgenic	Random integration	Xenotransplantation	[103]
Pig	Pdx1-Hes1	Transgenic	Random integration	Interspecies chimera	[118]
Pig	PERV	KO	CRISPR/Cas9	Xenotransplantation	[127,128]
Cattle	BLG	KO	ZFNs	Cattle milk modification	[131]
Cattle	BLG	KO	TALENs	Cattle milk modification	[132]
Goat	BLG (hLF)	KI	TALENs	Goat milk modification	[133]
Cattle	BLG (HSA)	KI	TALENs	Cattle milk modification	[134]
Pigs	MSTN	KO	ZFNs	Pig meat production, composition and quality	[136]
Goat	MSTN	KO	CRISPR/Cas9	Goat meat production, composition and quality	[137]
Goat	MSTN (fat-1)	KI	CRISPR/Cas9	Goat meat production, composition and quality	[138]
Pig	UCP1 (mouse UCP1)	KI	CRISPR/Cas9	Pig meat production, composition and quality	[139]
Pig	β-glucanase, xylanase, phytase	Transgenic	Random integration	Pig feed efficiency and environmental impact	[140]
Pig	CD163	KO	CRISPR/Cas9	Disease resistance to PRRSV	[141]
Pig	CD163 (SRCR 5 domain)	KI (hCD163L1 SRCR domain 8 homolog)	CRISPR/Cas9	Disease resistance to PRRSV	[142]
Pig	CD163 (SRCR 5 domain)	KO	CRISPR/Cas9	Disease resistance to PRRSV	[143]
Pig	FMDV-specific shRNA	Transgenic	Random integration	Disease resistance to FMDV	[144]
Cattle	NRAMP1	KI	CRISPR/Cas9	Disease resistance to tuberculosis	[146]
Cattle	SP110	KI	TALENs	Disease resistance to tuberculosis	[157]
Cattle	HORNED allele	KI (POLLED)	TALENs	Animal welfare	[148]
Pig	Human albumin	KI	CRISPR/Cas9	Bioreactor	[149]

KI, knockin; KO, knockout.
